# Current Understanding of Acute Bovine Liver Disease in Australia

**DOI:** 10.3390/toxins9010008

**Published:** 2016-12-26

**Authors:** Elizabeth Read, Jacqueline Edwards, Myrna Deseo, Grant Rawlin, Simone Rochfort

**Affiliations:** 1 Department of Economic Development, Jobs, Transport and Resources, Biosciences Research, AgriBio, the Centre for AgriBioscience, 5 Ring Road, Bundoora, Victoria 3083, Australia; jacky.edwards@ecodev.vic.gov.au (J.E.); myrna.deseo@ecodev.vic.gov.au (M.D.); grant.rawlin@ecodev.vic.gov.au (G.R.); simone.rochfort@ecodev.vic.gov.au (S.R.); 2 School of Applied Systems Biology, La Trobe University, Bundoora, Victoria 3086, Australia

**Keywords:** ABLD, hepatotoxicity, *Drechslera*, mycotoxicosis

## Abstract

Acute bovine liver disease (ABLD) is a hepatotoxicity principally of cattle which occurs in southern regions of Australia. Severely affected animals undergo rapid clinical progression with mortalities often occurring prior to the recognition of clinical signs. Less severely affected animals develop photosensitization and a proportion can develop liver failure. The characteristic histopathological lesion in acute fatal cases is severe, with acute necrosis of periportal hepatocytes with hemorrhage into the necrotic areas. Currently there are a small number of toxins that are known to cause periportal necrosis in cattle, although none of these have so far been linked to ABLD. Furthermore, ABLD has frequently been associated with the presence of rough dog’s tail grass (*Cynosurus echinatus*) and *Drechslera* spp. fungi in the pasture system, but it is currently unknown if these are etiological factors. Much of the knowledge about ABLD is contained within case reports, with very little experimental research investigating the specific cause(s). This review provides an overview of the current and most recently published knowledge of ABLD. It also draws on wider research and unpublished reports to suggest possible fungi and mycotoxins that may give rise to ABLD.

## 1. Introduction

Acute bovine liver disease (ABLD), formerly known as phytotoxic hepatitis, is a hepatotoxic disease principally affecting grazing beef and dairy cattle regardless of age, sex, or breed (Table 1). There have been two documented cases of mycotoxicoses in sheep with a similar epidemiology to ABLD, which implies ABLD may not be specific to cattle [1,2]. However, no further reports are documented, suggesting that if sheep are affected it is uncommon, presumably because of their differing grazing habits. This review will therefore focus on ABLD affecting cattle but much of the evidence presented is likely to be applicable to affected sheep.

ABLD affecting cattle is observed in the southeastern states of Australia (Victoria, Tasmania and parts of South Australia) [3,4], with at least one report of a possible occurrence in Western Australia in 2002 [5]. It has been a recognized condition since the 1950s though documented evidence from the early years is non-existent [4]. Lancaster et al. [6] collated findings from 15 reports not found in general circulation. Further documented ABLD events are contained in case reports, conference reports or newsletters shared between practicing veterinarians in relevant Australian states (Table 1) [3,6,7,8,9]. Accordingly, documented ABLD cases predominantly report clinical observations, findings of clinical and anatomical pathology, and suggestions that ABLD is caused by an unknown toxin. In addition to clinical findings, some case reports include limited epidemiological observations such as environmental and weather conditions, seasonality, and the presence or absence of plants of interest [7]. Interestingly, almost all reports have recorded the presence of senescent rough dog’s tail grass (*Cynosurus echinatus*) in the suspected paddock [6]. Rough dog’s tail grass was also present in the two reports of mycotoxicosis in sheep [1,2]. Consequently, risk factors for ABLD identified by state departments include long-standing dead or dry grass from the previous seasons, and the presence of rough dog’s tail grass [10,11].

Cases of ABLD are commonly reported in autumn/winter (April–July) almost every year, beginning around the time of the first rainfall and cooler temperatures after summer [10,11]. Climatic risk factors for ABLD include a minimum temperature of >12 °C, >4 mm rainfall with high humidity, and calm conditions for greater than days prior [4,7,11]. Furthermore, it is likely that additional cases of unrecognized or unreported ABLD also occur. This has resulted in speculation on the etiology and pathogenesis of the disease. The current advice to graziers includes avoiding putting cattle on high-risk paddocks, grazing out high-risk paddocks with sheep to reduce the amount of dry standing grass, cultivating high-risk paddocks, and grazing a small number of cattle on high-risk paddocks to test for toxicity.

## 2. Investigation and Diagnosis of ABLD

Clarke and Weaver [7] reported that the onset of clinical signs can occur as early as 12 h after apparent exposure. The clinical manifestation of ABLD consistently includes an initial drop in milk production, secondary photosensitization, and altered behavior such as seeking shade even on overcast days. Depression, pyrexia, loss of appetite, and agitation may be observed in some cattle. Acute cases may result in death before clinical signs are observed [7]. Upon necropsy, liver damage is evident and periportal necrosis is often observed histologically. Hemorrhaging into the necrotic periportal area and progressive hepatocyte damage may also be observed [3,7]. Serum biochemistry often reveals a marked elevation of glutamate dehydrogenase and aspartate transaminase activities and moderate increases in gamma-glutamyl transpeptidase activity, indicating liver damage [7]. Although a biochemistry pattern such as this is consistently observed when ABLD is diagnosed, it is not specific for the diagnosis of ABLD. Furthermore, although periportal necrosis is a characteristic feature of acute and fatal ABLD, the pathology that may be present in more chronic, sub-lethal cases has not been documented. It may be more difficult to differentiate from other hepatotoxicoses, and so incorrect diagnoses may be made. Additional documentation of histological features at different stages of toxicosis is required to better define ABLD.

While toxic hepatopathies are known to be caused by a range of toxic compounds, periportal necrosis is uncommon, which limits the differential diagnoses. The final diagnosis is currently based on the presence of characteristic acute periportal hepatocellular necrosis or consistent biochemistry changes and the exclusion of other differential diagnoses. The most common causes for hepatic necrosis in grazing cattle in southeastern Australia include blue/green algae poisoning, pithomycotoxicosis (facial eczema), and boobialla (*Myoporum tetrandrum*) poisoning [14,16,17]. While only boobialla causes periportal necrosis similar to ABLD, it has not been present when an outbreak of ABLD has been suspected. Therefore, a diagnosis of ABLD is achieved by testing available water sources for toxic forms of blue/green algae, a detailed examination of the paddock confirming the absence of other toxic plants, and verification of periportal necrosis in the liver. The presence of rough dog’s tail grass is commonly used for the initial diagnosis (before pathological investigation) of ABLD. However, the absence of rough dog’s tail grass does not exclude ABLD as a final diagnosis following sufficient pathological investigation. There are currently no methods for confirming ABLD on-farm without post-mortem examination, though ABLD can be strongly suspected based on epidemiology, clinical signs, biochemistry, and environmental examination.

The occurrence of ABLD is notoriously sporadic and unpredictable. Not all cattle on a property will be visibly affected and usually only a small number die, though large losses have also been observed [3,4,8,9]. Moreover, pastures that cause ABLD appear to be only transiently toxic, and farms may not experience another occurrence in the following weeks, months, or years. Despite this, multiple occurrences in the same year on the same property have been observed on farms when the first occurrence was early in the season (Mark Hawes, personal communication [18]).

Although ABLD has been recognized as a specific condition for 50 years, identification and initial diagnosis remain problematic. Initial investigation of clinical manifestations and pasture conditions is not enough to differentiate ABLD from other common illnesses, particularly in dairy cattle (unpublished data). Furthermore, testing blood and other body fluids does not specifically indicate ABLD; therefore, no specialized surveillance methods are available [7]. A thorough clinical examination, epidemiological investigation, and comprehensive necropsy will often result in a diagnosis. However, this takes time and is dependent on the findings of the initial investigation and the farmer’s support for further investigation. A farmer may not support further investigation into the death of a small number of cows if they do not consider the deaths to be a significant loss. These factors make it particularly difficult to investigate possible ABLD occurrences.

## 3. Suspected Causes of ABLD

At locations and times that ABLD has been investigated, the pasture commonly contains senescent rough dog’s tail grass [3,5,8]. However, rough dog’s tail grass is found worldwide and is not reported to cause illness [19]. Lancaster et al. [6] found that rough dog’s tail grass harvested from an affected property and fed to cattle on two occasions resulted in no ill effects. The first feeding trial was conducted in Tasmania with calves and remains unpublished (cited in [6]), while the second was conducted in Victoria in 2003. In the latter, rough dog’s tail grass was harvested from six properties that had experienced ABLD in the previous two years. The grass was collected in August (winter), approximately six weeks after the perceived ‘danger period’ for ABLD. In the trial, oats and rough dog’s tail were inoculated with spores of *Drechslera biseptata* and incubated for seven days. The inoculated grass/oats were fed to young bulls and a fungal broth of *D. biseptata* was additionally administered by stomach tube. No ill effects were observed, suggesting the grass without the specific conditions needed for toxicity is unlikely to cause ABLD. The significance of *D. biseptata* will be discussed later in this review.

Fungal infections of the grass are now the suspected cause, as the epidemiology of ABLD has similar characteristics to other illnesses caused by mycotoxins [3,17]. Aside from the usual acute and chronic toxicoses, mycotoxins can alter feed intake, production (milk production or growth rate), nutrient utilization, reproduction, and product quality (including residues in milk and meat) [20,21,22].

## 4. Some Known Mycotoxins Affecting Cattle

Some fungi may be present as endophytes within vegetative plant material, for example *Epichloë festucae* var. *lolii* found in perennial ryegrass (*Lolium perenne*). Reed et al. [23] reported that *E. festucae* var. *lolii* provides resources for the grass, enabling its survival under conditions it would not normally survive. However, *E. festucae* var. *lolii* also produces alkaloids that cause toxicosis of ruminants and other grazing livestock. Toxicosis commonly results in neurological signs such as tremors and staggering, as well as loss in production and occasional deaths [24]. To date there has been no published research into the presence of specific endophytic fungi associated with rough dog’s tail grass, nor are endophytic fungi currently associated with liver damage in cattle.

*Aspergillus* spp. and *Pithomyces chartarum* are the most noteworthy fungi when investigating hepatotoxic mycotoxin production [22,24,25]. *Aspergillus flavus* and *Aspergillus parasiticus* are the most common species in agriculture which produce aflatoxins. These fungi are commonly found in ground nut or peanut meal but can also be present in silage and high-moisture feeds [22,24]. Aflatoxin exposure causes liver damage, resulting in jaundice, photosensitization, diarrhea, anorexia, depression and eventual death in livestock [26,27]. However, *Aspergillus* spp. and aflatoxins have not been found to cause liver pathology similar to ABLD.

*Pithomyces chartarum* is a saprophytic fungus found on dead or dry pasture [28]. Spores of *P. chartarum* contain sporidesmin, and ingestion of sufficient spores results in facial eczema in sheep and cattle. Comparable to aflatoxin poisoning and ABLD, early facial eczema in cattle results in diarrhea, anorexia, depression, and eventually jaundice, photosensitization and death [29]. Furthermore, acutely affected sheep and cattle can die suddenly without suffering photosensitization, and periportal hepatocytes may be affected [29].

The epidemiology of facial eczema is also strikingly similar to ABLD. Christensen and Tuite [28] and Riet-Correa et al. [29] both noted that *P. chartarum* predominates on dead grasses during periods of rainy or overcast days, with high relative humidity, and temperatures close to 20 °C. As such, facial eczema occurs during late summer and autumn. However, *P. chartarum* is not considered to cause ABLD since the type of periportal necrosis associated with ABLD is not consistent with hepatic pathological changes seen in facial eczema. Furthermore *P. chartarum* spores have not been detected, either at all or in numbers consistent with causing disease, in pastures associated with outbreaks of ABLD. Thus, ABLD is most likely caused by a currently unidentified fungal pathogen/toxin.

## 5. Possible Fungi Associated with ABLD

With regards to possible fungal contaminants of rough dog’s tail grass and their role in ABLD, some published and unpublished data is available. In 2006, samples of rough dog’s tail grass were collected within a few days of outbreaks of ABLD and investigated for the presence of possible fungal contamination (Ian Pascoe, unpublished work [15]). The most common fungal species identified were *Colletotrichum graminicola* and *Drechslera* sp. aff. *siccans*. *Drechslera biseptata* and *Colletotrichum* sp. aff. *coccodes* were also identified, but were less common. *Colletotrichum graminicola* was identified in a majority of the samples collected, but has not been reported to produce mycotoxins, and therefore is not thought to cause ABLD. Since the 2006 investigation, both *D*. sp. aff. *siccans* and *D. biseptata* have been identified in samples collected during outbreaks of ABLD in 2013, 2014 and 2015 [30]. *D*. sp. aff. *siccans* has been consistently more abundant than *D. biseptata* and is therefore the most likely candidate for toxin production.

### 5.1. Drechslera sp. as a Source of Mycotoxin Causing ABLD

Long-term culturing of *D*. sp. aff. *siccans* has been unsuccessful, while cultures of *D. biseptata* are more stable, making *D. biseptata* more suitable for toxicity testing. Consequently, the only relevant published data on the toxicity of *Drechslera* spp. to cattle is by Lancaster et al. [6] and Aslani et al. [14]. As stated earlier, Lancaster et al. [6] fed rough dog’s tail grass inoculated with *D. biseptata* spores to bulls, with and without additional administration of a broth containing *D. biseptata* via stomach tube. No ill effects were observed during five days of monitoring or in the subsequent necropsy. Consequently, *D. biseptata* is considered to be an unlikely source of the toxin under these conditions.

Later, Aslani et al. [14] extracted the spores and mycelium of *D. biseptata* using various solvents and tested these in vitro with clone 9 rat hepatocytes. A methanolic extract of pasture samples, collected in 2003, was also tested. Cells were treated with: water or hexane extracts of mycelium or spores (least degeneration); methanol extract of mycelium; methanol fractions of hexane, water and ethyl acetate extracts of mycelium; methanol extract of whole fungal culture (various concentrations); and the methanol extract of rough dog’s tail pasture samples. Hepatocyte degeneration was observed in all tests by examining morphological changes of the cells. Furthermore, the methanol extract of the whole fungal culture was found to have a dose-dependent effect. This suggests there are potential toxins present in these extracts. However, a potential toxin was only putatively identified, and an effect in vitro using rat hepatocytes does not indicate a similar effect will occur in in vivo bovine hepatocytes. Further research into the involvement of *Drechslera* spp. and their toxins is required.

### 5.2. Mycotoxins Produced by Drechslera spp.

*Drechslera* spp. are documented as important, world-wide infectious agents of various plants [31,32,33,34,35]. They have been identified as the cause of toxicosis and illness in humans [31,36,37,38] and animals [31,37,38,39], under various circumstances. Different *Drechslera* spp. have been found to produce a variety of toxins that affect plants, other fungi, bacteria, and potentially animals.

Given that *Drechslera* spp. are predominantly plant pathogens, many of the toxins produced have been characterized for their effect on plants, not on other organisms. For example, *Drechslera siccans*, a pathogen of ryegrass, has been shown by Evidente et al. [40] to produce drazepinone, which has herbicidal activity. Earlier, Sugawara et al. [33] found *Drechslera maydis* and *Drechslera sorghicola* both produce phytotoxic sesterterpenoids belonging to the ophiobolin family of compounds.

**Figure 1:**
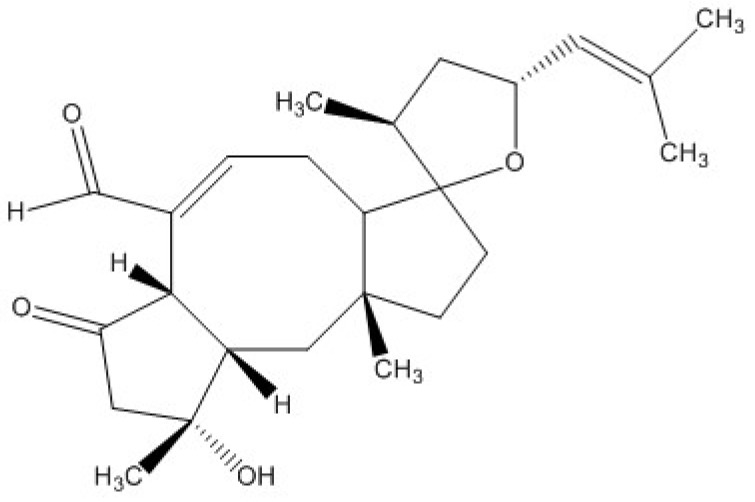
Ophiobolin A.

Strobel et al. [32] summarized that there are a number of different *Drechslera* spp. that produce ophiobolins. Recently, researchers have shown that ophiobolins are cytotoxic to various mammalian cells. Bencsik et al. [41] found that ophiobolin A (Figure 1) inhibited the mobility of porcine spermatozoa, and damaged the mitochondria in these cells by changing the mitochondrial membrane potential, even at sub-lethal doses. Similarly, Bury et al. [42] found that ophiobolin A caused cytoskeletal changes, interfered with Ca^2+^ and K^+^ channel activity, and induced paraptosis-like cell death in human glioblastoma cells. Unfortunately, there is limited published evidence of ophiobolin toxicity in vivo; therefore, the potential toxicity of ophiobolins to cattle is unknown.

**Figure 2:**
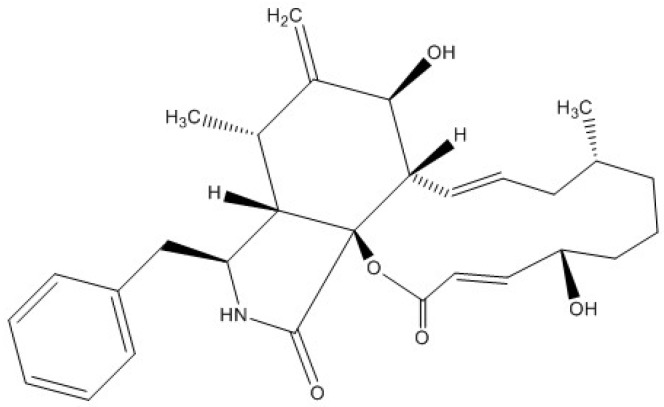
Cytochalasin B.

The potentially causative toxin that Aslani et al. [14] isolated from toxic extracts of *D. biseptata* was putatively identified as being cytochalasin-like due to its mass spectral profile. Capio et al. [43] identified cytochalasin B (Figure 2) as a possible toxic compound produced by *D. wirreganensis* and *D. campanulata*. Correspondingly, Schneider et al. [39] implicated *D. campanulata* in the poisoning of goats, although the toxic principle was not identified. Furthermore, Collett et al. [44] observed mycotoxicosis in rats fed cultures of *D. campanulata*, but again the specific toxin was not identified. Earlier research by Smith et al. [45] and Ridler and Smith [46] showed cytochalasin B induced morphological changes in in vitro cultures of human lymphocytes. Additionally, Tanenbaum [47], Kim et al. [48] and Zhang et al. [49] demonstrated that cytochalasins are produced by a variety of fungi and are biologically active in many ways, including: phytotoxicity, anti-microbial activity, cytotoxicity, capping of actin filaments, and inhibition of HIV-1 protease 2. This suggests cytochalasins produced by *Drechslera* spp. could be potential toxin candidates for ABLD.

## 6. Likelihood of *Drechslera* spp. to Cause ABLD

Rough dog’s tail grass, present as dry, senescent grass during autumn, is not preferentially grazed by cattle when fresh, green pasture is also available. However, *D. biseptata* is currently only associated with rough dog’s tail and no other pasture species. Commonly, early growth of fresh pasture is found around the base of dry senescent rough dog’s tail grass (Mark Hawes, personal communication [18]). As such, cattle may be inadvertently ingesting some rough dog’s tail grass with young green grass, thus ingesting associated toxic fungi. Since rough dog’s tail is not preferentially grazed, the amount of toxin ingested is likely to be limited in this scenario. This suggests the toxin is either particularly potent, or there is a significant amount of toxic *Drechslera* spp. present on ingested rough dog’s tail grass.

Alternatively, *Drechslera* spp. may be producing toxic spores that can be transferred between rough dog’s tail grass and new pasture growth and thus ingested by cattle. Conditions favoring *Drechslera* spp. sporulation include changes in relative humidity combined with decreasing temperatures [50,51]. Troutt and Levetin [52] reported *Drechslera* spores were common when there were warmer afternoon temperatures. *Drechslera* species have been found to preferentially sporulate at ~21 °C with light intensities near UV under laboratory conditions [50,51,53]. When consideration is given to the timing of toxic ABLD events (increasing rainfall with a change from warm to cold weather), autumn would be the ideal time for fungal sporulation [7]. An autumn occurrence is consistent with the findings of Burch and Levetin [54] who found spore densities in the atmosphere were highest around midday during autumn. Furthermore, other spore-related mycotoxicoses such as facial eczema often occur during autumn [55].

Currently, the experimental evidence suggests *Drechslera* spp. infecting rough dog’s tail is the principle source of ABLD toxin. Aslani et al. [14] found spores only had limited cytotoxicity; thus, spores may also contain ABLD toxin, but at a much lower concentration. Consequently, ingestion of either or both mycelium or spores may cause ABLD and the grazing habits of individual cattle would likely affect the concentration of ABLD toxin ingested. As with most poisonings, the concentration of toxin ingested is likely to cause the diversity in clinical signs commonly observed for ABLD.

## 7. Conclusions

Autumn climatic conditions may stimulate the production of fungal toxins or toxic spores which the cattle are exposed to when grazing infected grass. Contaminants of senescing rough dog’s tail grass could be transferred to nearby palatable pasture via natural processes or by mechanical means. However, the specific toxin(s) and their source remain conjectural and their stability in the environment is unknown. Therefore, even though the concentration of the causative toxins will be greater in feed source materials, this may be a situation where the examination of tissues from animals that have died may provide some insight. It is hypothesized that any suspicious compounds detected may provide an indication of the nature of the toxin(s). Furthermore, the presence of rough dog’s tail grass and *Drechslera* spp. during an outbreak of ABLD remains suspicious. Consequently, it is hypothesized that *Drechslera* spp. associated with rough dog’s tail grass may be the source of the toxin(s) of interest.

## Figures and Tables

**Table 1:** Published investigations of ABLD outbreaks.

Authors [Reference]	Year	Publication Type	Breed/Age	No. Affected
Clarke and Weaver [7]	2001	Conference proceeding	Beef and dairy/various ages	30+
Allen and Graydon [5]	2002	Newsletter case report	-	-
Winterbottom [8]	2002	Newsletter case report	Dairy/various ages	9/27 34/80
Jubb [3]	2003	Newsletter case report	Dairy/various ages	34/270 4/50
Gunn and Clarke [12]	2003	Newsletter case report	-	-
Kelly et al. [13]	2003	Conference proceeding	-	-
Dickason [4]	2006	Newsletter case report	Hereford cows	3
Jubb [9]	2006	Newsletter case report	Hereford and Angus cows 10 month old Fresian heifers	7/25 1/35
Lancaster et al. [6]	2006	Short communication	9 month old Fresian bulls	0/4
Aslani et al. [14]	2006	Research article	-	-
Pascoe [15]	2006	Fungal consultancy report	-	-
